# Supplementary material for: Self-organization of modular activity in immature cortical networks
Source: Nat Commun. 2024 May 21;15:4145. doi: 10.1038/s41467-024-48341-x (PMC11109213; doi:10.1038/s41467-024-48341-x)
Supplement: Supplementary file 3 — Description of Additional Supplementary Files [file 41467_2024_48341_MOESM3_ESM.pdf]

File name: Supplementary Movie 1
Description: Uniform optogenetic stimulation of developing V1 reliably evokes modular cortical activity. Modular activity emerges in response to uniform cortical stimulation, with multiple coactive local regions distributed across several millimeters of cortex.
