# Supplementary material for: Self-organization of modular activity in immature cortical networks
Source: Nat Commun. 2024 May 21;15:4145. doi: 10.1038/s41467-024-48341-x (PMC11109213; doi:10.1038/s41467-024-48341-x)
Supplement: Supplementary file 5 — Reporting Summary [file 41467_2024_48341_MOESM5_ESM.pdf]

nature portfolio

Corresponding author(s): Gordon Smith

Last updated by author(s): Mar 27, 2024

Reporting Summary

Nature Portfolio wishes to improve the reproducibility of the work that we publish. This form provides structure for consistency and transparency in reporting. For further information on Nature Portfolio policies, see our Editorial Policies and the Editorial Policy Checklist.

Statistics

For all statistical analyses, confirm that the following items are present in the figure legend, table legend, main text, or Methods section.

n/a	Confirmed	
☐	☒	The exact sample size (n) for each experimental group/condition, given as a discrete number and unit of measurement
☐	☒	A statement on whether measurements were taken from distinct samples or whether the same sample was measured repeatedly
☐	☒	The statistical test(s) used AND whether they are one- or two-sided<br>*Only common tests should be described solely by name; describe more complex techniques in the Methods section.*
☒	☐	A description of all covariates tested
☐	☒	A description of any assumptions or corrections, such as tests of normality and adjustment for multiple comparisons
☐	☒	A full description of the statistical parameters including central tendency (e.g. means) or other basic estimates (e.g. regression coefficient) AND variation (e.g. standard deviation) or associated estimates of uncertainty (e.g. confidence intervals)
☐	☒	For null hypothesis testing, the test statistic (e.g. F, t, r) with confidence intervals, effect sizes, degrees of freedom and P value noted<br>*Give P values as exact values whenever suitable.*
☒	☐	For Bayesian analysis, information on the choice of priors and Markov chain Monte Carlo settings
☐	☒	For hierarchical and complex designs, identification of the appropriate level for tests and full reporting of outcomes
☐	☒	Estimates of effect sizes (e.g. Cohen's d, Pearson's r), indicating how they were calculated

Our web collection on statistics for biologists contains articles on many of the points above.

Software and code

Policy information about availability of computer code

Data collection	Widefield epifluorescence calcium imaging was collected with μManager (2.0.0-gamma1, Edelstein et al., 2010), optogenetic stimulation was delivered using PolyScan2 (1.2.2, Mightex), and visual stimulation was delivered with Psychopy (2020.2.8, Peirce 2008).
Data analysis	Data was analyzed in Python (version 3.8) using open source libraries (Numpy 1.22.3, SciPy 1.7.3, Scikit-learn 1.0.2) and custom algorithms. Code used to run computational simulations is available on GitHub at: https://github.com/SmithNeuroLab/uniform_opto. DOI: 10.5281/zenodo.10892426

For manuscripts utilizing custom algorithms or software that are central to the research but not yet described in published literature, software must be made available to editors and reviewers. We strongly encourage code deposition in a community repository (e.g. GitHub). See the Nature Portfolio guidelines for submitting code & software for further information.

Data

Policy information about availability of data

All manuscripts must include a data availability statement. This statement should provide the following information, where applicable:

- Accession codes, unique identifiers, or web links for publicly available datasets
- A description of any restrictions on data availability
- For clinical datasets or third party data, please ensure that the statement adheres to our policy

All source data for figures are provided with this paper. Due to size limitations, all original raw data (imaging files) are available from the corresponding author upon request.

Research involving human participants, their data, or biological material

Policy information about studies with human participants or human data. See also policy information about sex, gender (identity/presentation), and sexual orientation and race, ethnicity and racism.

Reporting on sex and gender	N/A
Reporting on race, ethnicity, or other socially relevant groupings	N/A
Population characteristics	N/A
Recruitment	N/A
Ethics oversight	N/A

Note that full information on the approval of the study protocol must also be provided in the manuscript.

Field-specific reporting

Please select the one below that is the best fit for your research. If you are not sure, read the appropriate sections before making your selection.

☒ Life sciences ☐ Behavioural & social sciences ☐ Ecological, evolutionary & environmental sciences

For a reference copy of the document with all sections, see nature.com/documents/nr-reporting-summary-flat.pdf

Life sciences study design

All studies must disclose on these points even when the disclosure is negative.

Sample size	No statistical methods were used to predetermine sample sizes, but our sample sizes are similar to those reported in previous publications (White et al., 2001; Smith et al., 2018; Mulholland et al., 2021).
Data exclusions	Data from animals that failed to show significant optogenetic responses over a cortical surface area greater than 1mm^2 were excluded from this study, as it could not be concluded that the cortical network was being stimulated with a uniform input.
Replication	Experiments were repeated across multiple animals (n=8 for the uniform stimulation experiments, n=7 for the structured stimulation, and n=3 for the pharmacological experiments), and both within-animal and across-animal repetition summary statistics were used when appropriate. All imaged animals are reflected in the data (except as noted above). We were able to reproduce results across animals, except in rare circumstances, in which case the number of animals or experiments that failed to produce significant results are highlighted in the figure and/ or text.
Randomization	No attempt was made to randomize experimental groups. All control experiments come from within-animal controls, and paired statistics were used when appropriate.
Blinding	Blinding as not possible in this study as it was not possible to prevent the pattern(s) of optogenetic stimulation from being known to the experimenter, and no attempt was made to blind experimenters during data collection or analysis. Note that in all cases experimental conditions were compared to within-animal controls.

Reporting for specific materials, systems and methods

We require information from authors about some types of materials, experimental systems and methods used in many studies. Here, indicate whether each material, system or method listed is relevant to your study. If you are not sure if a list item applies to your research, read the appropriate section before selecting a response.

Materials & experimental systems

n/a	Involved in the study
☒	☐ Antibodies
☒	☐ Eukaryotic cell lines
☒	☐ Palaeontology and archaeology
☐	☒ Animals and other organisms
☒	☐ Clinical data
☒	☐ Dual use research of concern
☒	☐ Plants

Methods

n/a	Involved in the study
☒	☐ ChIP-seq
☒	☐ Flow cytometry
☒	☐ MRI-based neuroimaging

Animals and other research organisms

Policy information about studies involving animals; ARRIVE guidelines recommended for reporting animal research, and Sex and Gender in Research

Laboratory animals	Ferrets age postnatal day 23-29 were used in this study.
Wild animals	No wild animals were used in this study.
Reporting on sex	Both male and female ferrets were used in this study, and no sex-based segregation or analysis was conducted for this study.
Field-collected samples	No field-collected samples were used in this study.
Ethics oversight	All experimental procedures were approved by the University of Minnesota Institutional Animal Care and Use Committee and were performed in accordance with guidelines from the US National Institutes of Health

Note that full information on the approval of the study protocol must also be provided in the manuscript.
